# Temperature in Surgical Cases

**Published:** 1874-04

**Authors:** 


Temperature in Surgical Cases.—Dr. Joseph Bell, of Edinburgh, in a paper on surgical cases in relation to temperature, lays down the following axioms:

1. Suppuration, even very profuse, does not necessarily imply any great rise in temperature, so long as it is not putrid. 2. Fœtor or putrefaction of suppuration always induces a rise in temperature. 3. A high temperature, lasting for more than three or four days after the injury or operation, indicates mischief impending, such as sloughing or abscess. 4. The temperature generally gives warning a day, or even two days, before the pulse.—*Richmond and Louisville Journal.*

